# From Classroom to Clinic: Simulation-Based Psychiatry Teaching for Medical Students

**DOI:** 10.1192/bjo.2026.11288

**Published:** 2026-06-30

**Authors:** Sharon Rajesh, Leanne Tozer

**Affiliations:** University of Plymouth, Plymouth, United Kingdom

## Abstract

**Aims:**

Undergraduate medical students frequently have limited exposure to acutely unwell psychiatric patients, resulting in reduced confidence and practical competence in psychiatric history-taking and Mental State Examination (MSE). This study describes the development, delivery, and evaluation of a single-site simulation-based psychiatric teaching programmeaimed to deliver realistic and structured simulation experiences. The primary aims were to enhance students’ skills in psychiatric assessment, differential diagnosis, and initial management planning, whilst also strengthening understanding of mental health law and pharmacological treatment.

**Methods:**

A three-hour simulation session was delivered to small groups of third- and fourth-year medical students during their psychiatry placement, with 3–5 students per session. Each student conducted a focused psychiatric history and MSE with a simulated acutely unwell patient, followed by presentation of findings, differential diagnosis, and an immediate management plan. Sessions incorporated structured multi-source feedback from peers, facilitators, and the simulated patient actor. Pre-and post-session questionnaires measured baseline and post-session self-efficacy, knowledge, and learner perceptions. Comparative placement evaluation data were reviewed between cohorts with and without embedded simulation teaching. Session design was informed by experiential, constructivist, and adult learning principles, with careful attention to cognitive load and psychological safety.

**Results:**

Students demonstrated substantial improvements in self-efficacy across all assessed domains. Pre-session ratings averaged 2–3/5 for psychiatric history-taking, MSE, and communication with acutely unwell patients, increasing to 4–5/5 post-session, representing a 90% increase in self-reported confidence. Cohorts with embedded simulation reported consistently higher satisfaction with their psychiatry pathway than cohorts without simulation, including higher ratings for overall week quality (mean 4.3–4.5 vs 3.0), usefulness of the introduction (mean 4.7–5.0 vs 3.9), and feedback in clinical reasoning (mean 4.0–4.7 vs 3.0). Qualitative feedback described the session as “the most helpful thing of the placement”,highlighting realistic acting, varied scenarios, and the value of structured feedback within a supportive, non-judgemental environment. Following positive evaluation, the programme progressed from a pilot to integration within the University of Plymouth undergraduate psychiatry pathway, with planned expansion to affiliated hospitals.

**Conclusion:**

Simulation-based psychiatric teaching effectively addresses gaps in undergraduate exposure to acutely unwell patients, improving confidence, practical skills, and reflective practice. This sustainable and scalable model demonstrates how locally developed simulation can enhance placement quality and achieve wider institutional adoption, offering an innovative and transferable approach to undergraduate psychiatry education.

